# YOLOv8-DMC: Enabling Non-Contact 3D Cattle Body Measurement via Enhanced Keypoint Detection

**DOI:** 10.3390/ani15182738

**Published:** 2025-09-19

**Authors:** Zhi Weng, Wenwen Hao, Caili Gong, Zhiqiang Zheng

**Affiliations:** 1 College of Electronic Information Engineering, Inner Mongolia University, Hohhot 010021, China; wzhi@imu.edu.cn (Z.W.); hww@mail.imu.edu.cn (W.H.); eegcl@imu.edu.cn (C.G.); 2 State Key Laboratory of Reproductive Regulation & Breeding of Grassland Livestock, Hohhot 010021, China; 3 Research Base for Dairy Farming Engineering and Full Mechanization of Equipment, Ministry of Agriculture and Rural Affairs, Hohhot 010018, China

**Keywords:** cattle body measurement, YOLOv8-DMC, keypoint detection, depth completion, 3D point cloud reconstruction, precision livestock farming, non-contact livestock monitoring

## Abstract

Accurate and non-contact measurement of cattle body dimensions is essential for precision livestock management. This study presents YOLOv8-DMC, a lightweight deep learning model optimized for anatomical keypoint detection in side-view images of cattle. The model integrates three attention modules—DRAMiTransformer, MHSA-C2f, and CASimAM—to improve robustness under occlusion and lighting variability. Following keypoint prediction, a 16-neighborhood depth completion and pass-through filtering process are applied to generate clean, colored point clouds. This enables precise 3D localization of keypoints by matching them to valid depth values. The model achieves $AP_{@0.5}$ of 0.931 and AP@[0.50:0.95] of 0.868 on a dataset of over 7000 images, improving baseline accuracy by 2.14% and 3.09%, respectively, with only 0.35 M additional parameters and 0.9 GFLOPs in complexity. For real-world validation, strictly lateral-view RGB-D images from 137 cattle were collected, with ground-truth manual measurements. Compared with manual measurements, the average relative errors are 2.43% for body height, 2.26% for hip height, 3.65% for body length, and 4.48% for cannon circumference. The system supports deployment on edge devices, providing an efficient and accurate solution for 3D cattle measurement in real-world farming conditions.

## Simple Summary

Accurately measuring the body size of cattle is essential for health monitoring and effective farm management. Traditional manual methods are time-consuming, labor-intensive, and may cause stress to the animals. In this study, we developed an automated, non-contact system that combines image-based keypoint detection with 3D depth data to estimate key body measurements. A lightweight deep learning model was enhanced to better detect anatomical landmarks even under poor lighting or partial occlusion. The system also processes depth information to create a clear 3D model of the animal, allowing reliable measurement of traits such as height, length, and limb circumference. Designed to run on low-power devices, this method offers a practical solution for real-time cattle monitoring in real-world farm environments.

## 1. Introduction

Precision livestock farming is an inevitable trend in the development of animal husbandry, and accurate body measurement is a critical component of this approach [1,2]. Body dimensions are closely related to carcass composition and genetic improvement in livestock. For example, studies on pigs have shown that body size parameters strongly correlate with carcass traits, highlighting the importance of accurate measurement for breeding evaluation and selection [3,4]. Currently, body measurements in cattle rely heavily on labor-intensive manual methods. These methods are prone to inaccuracy and may compromise animal welfare by inducing stress-related behaviors. Therefore, developing non-contact methods for cattle body measurement is of great significance [5,6,7].

In studies based on two-dimensional (2D) images, Zhang et al. developed a non-contact method for measuring sheep body size. They applied machine learning techniques to extract the body contour and then identified key anatomical landmarks to obtain body measurements. However, this method has certain limitations. When the background is complex, the accuracy of contour extraction can be affected due to the sensitivity of image clustering algorithms to environmental noise [8]. In addition, 2D image-based methods require strict calibration and can only measure limited body dimensions, lacking accuracy in three-dimensional (3D) spatial data. To overcome these issues, some researchers have used Kinect depth cameras to capture depth data [9,10,11]. In studies [12,13], multiple depth cameras were employed to collect point clouds for 3D reconstruction, which is a common approach. Poisson surface reconstruction algorithms were then applied to generate complete 3D models and extract body measurements [14].

In the field of three-dimensional (3D) data analysis, an increasing number of researchers have employed point cloud technology to measure livestock body dimensions. Depth cameras are used to collect point cloud data of animals, and through techniques such as point cloud segmentation and registration, a 3D contour model of the animal is constructed. By identifying key measurement points directly on the point cloud, body size parameters can be calculated in the world coordinate system. Guo et al. [15] developed an interactive software, LSSA_CAU version 1.7, for estimating livestock body length based on 3D point cloud data. This tool provides a semi-automatic workflow for loading, rendering, segmenting, posture normalization, and measuring the full-body surface point cloud of animals. However, it assumes that the cattle or pigs are standing with similar postures and facing forward. Huang et al. [16] utilized a classical point cloud dataset from the ShapeNet repository to train a KD-network using deep learning techniques. A LiDAR (Light Detection and Ranging) sensor was employed to perceive point cloud data (PCD), extract cattle contours, and identify candidate surface regions based on mean and Gaussian curvature. After computing feature histograms of the surface, the center of the feature area was identified, allowing for final body size estimation. Currently, most point cloud-based body measurement methods rely on complete, registered point clouds of the entire animal. Some studies have explored the relationship between body size parameters and genome-wide association studies (GWAS) [17], using keypoint-based measurement methods that extract landmarks from the full-body point cloud of dairy cows [18]. This approach reduces the number of processing steps and ensures data continuity. However, Full-body point clouds often contain more than 150,000 points, and differences in point density and occlusion across body regions further increase preprocessing and registration costs. This substantial computational overhead may also introduce interference from irrelevant body parts, and the computational load is high [19,20].

Although progress has been made, relatively few studies have focused specifically on automated cattle body measurement. Existing approaches still face challenges such as sensitivity to complex backgrounds in 2D methods, heavy computational overhead in full-body point cloud reconstruction, and reduced robustness under posture variation or occlusion. These shortcomings limit their applicability in real farming environments where measurement needs to be efficient, accurate, and non-invasive. Against this background, the present work introduces a lightweight deep learning approach that combines 2D keypoint prediction with 3D point cloud processing. By addressing both keypoint localization accuracy and point cloud reliability, our method aims to overcome unresolved challenges in cattle measurement and contribute a practical solution for precision livestock farming.

In this study, we propose an automated, non-contact cattle body measurement system that integrates both two-dimensional (2D) and three-dimensional (3D) data. To address the challenges of keypoint extraction and insufficient point cloud accuracy in cattle body measurement, we first configured the parameters of the Intel RealSense D455i camera (Intel Corporation, Santa Clara, CA, USA) to establish a one-to-one correspondence between RGB pixels and depth map values. An improved deep learning model based on YOLOv8n-pose was then developed to enable high-precision prediction of key anatomical landmarks. The raw point cloud data collected by the camera were subsequently preprocessed using depth completion based on a 16-neighbor mean algorithm and a pass-through filtering method. Finally, the processed point cloud was fused with the predicted RGB image to reconstruct a 3D model of the animal, from which accurate body measurements were computed.

## 2. Materials and Methods

### 2.1. Data Collection

In this study, cattle were only imaged during routine herd management without any handling, invasive procedures, or additional interventions. Therefore, no animal care or ethics committee approval was required.

**Figure 1:**
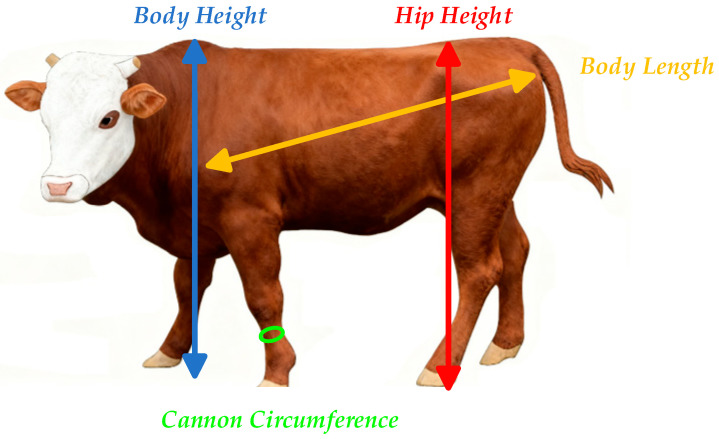
Annotations of key body measurement indicators in a cattle image.

In this study, Chinese Yellow Cattle were selected as the research subjects, and a dataset of key anatomical landmarks for body measurement was constructed. Data collection was conducted in 2023 at the Shengquan Animal Husbandry Ranch, located in Ongniud Banner, Chifeng City, Inner Mongolia Autonomous Region, China. A total of 137 lateral images of individual cattle were captured using an Intel RealSense D455i camera (Intel Corporation, Santa Clara, CA, USA), positioned approximately 1.5 m from the side of each animal. The camera was configured to simultaneously capture color and depth images at a resolution of 848 × 480 pixels, with a one-to-one correspondence between RGB pixels and depth values. To enhance the generalization ability of the proposed model, two publicly available datasets were incorporated. The first is a dataset developed by Ruchay et al. in the study entitled “Accurate Body Measurement of Live Cattle Using Three Depth Cameras and Non-Rigid 3D Shape Recovery” [21]. The second is a subset of the COCO dataset, consisting of side-view images of cattle in various poses. After an initial screening process, redundant images were removed, and representative samples were retained for annotation of six key anatomical landmarks associated with body measurements, including withers height, hip height, body length, and cannon circumference. Manual labeling was conducted using the Labelme 5.5.0 tool [22]. The measurement definitions were as follows: withers height was defined as the vertical distance from the highest point of the withers to the ground; body length as the straight-line distance from the point of the shoulder to the posterior edge of the ischium; hip height as the vertical distance from the highest point of the back at the front edge of the hip to the ground; and cannon circumference as the girth of the narrowest part of the forelimb cannon bone. To enhance the robustness and generalization ability of the model under complex environmental conditions, various data augmentation techniques were applied, including image translation, Gaussian noise addition, brightness adjustment, gamma contrast modification, and random occlusion generation to simulate realistic visual obstructions. Figure 1 illustrates an example of the captured cattle image with detailed annotations of key body measurement indicators.

The final dataset was divided into training, validation, and test sets in a 7:2:1 ratio, consisting of 4954, 1415, and 708 images, respectively. The division of the dataset is presented in Table 1.

### 2.2. Detection of Key Measurement Points in Cattle

#### 2.2.1. YOLOv8 Keypoint Detection Algorithm

In this study, the YOLOv8 algorithm from the YOLO series was adopted as the base model for keypoint detection. Specifically, YOLOv8-pose introduces a dedicated regression branch tailored for keypoint estimation tasks. The overall model architecture consists of three main components: the backbone, which extracts multi-scale image features; the neck, which fuses hierarchical features to enhance spatial representation; and the pose head, which predicts both object detection outputs and the coordinates and confidence scores of the keypoints. This structure supports end-to-end training and enables efficient joint inference for object detection and pose estimation, making it well-suited for applications that require both high accuracy and real-time performance [23].

Considering the constraints of real-world ranch environments—such as inference speed and deployment cost—we selected the lightweight YOLOv8n version. This variant allows for fast and accurate cattle body measurement and is compatible with edge computing devices. Keypoint detection is primarily used to localize anatomically significant parts of target objects and is widely applied in tasks such as human pose estimation and animal behavior analysis. In the present study, the target is cattle, and the keypoints are concentrated around anatomically representative regions visible from the side view. These points exhibit a well-defined structural distribution, making them suitable for modeling and prediction using structured keypoint detection methods [24,25].

#### 2.2.2. DRAMiTransformer Module for Shallow Attention

During on-site deployment in actual ranch environments, certain monitored areas contained occluding structures such as small fences and metal bars, which led to partial loss of keypoint information on the cattle body—particularly in regions such as the shoulders and legs, which are more prone to occlusion. Additionally, the presence of fences was often accompanied by changes in viewing angles and blurred occlusion boundaries, further reducing the accuracy of keypoint detection in these challenging scenarios.

**Figure 2:**
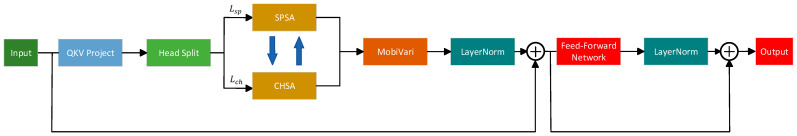
Structure of the DRAMiTransformer module.

To enhance the model’s global representation capability of cattle body structures and improve robustness under occlusion, a lightweight Transformer module—DRAMiTransformer—was introduced in this study [26]. Originally proposed for image restoration tasks such as denoising, deblurring, and super-resolution reconstruction, DRAMiTransformer offers a favorable trade-off between performance and computational efficiency, significantly reducing the number of parameters and computational cost while maintaining high accuracy. Given the practical demands of real-time deployment on ranches, where keypoint detection must be both precise and efficient, DRAMiTransformer presents a highly adaptable solution. This module consists of two attention branches: Spatial Reciprocal Attention and Channel Reciprocal Attention. The former replaces conventional self-attention with reciprocal attention to approximate global modeling while reducing computational complexity. The latter captures interdependencies among feature channels, thereby enhancing the model’s ability to learn semantic representations. The outputs from both branches are fused through an attention mixing mechanism, followed by a feed-forward network (FFN) to improve nonlinear feature representation. The overall structure of DRAMiTransformer is illustrated in Figure 2.

To enhance the modeling capability of high-resolution features in the shallow prediction branch (P3/8), the DRAMiTransformer module was integrated into the head of the YOLOv8 architecture. Specifically, it was inserted at the 15th layer—immediately after the C2f operation on the P3 feature map. This integration allows the model to better capture both local and global contextual relationships, thereby improving its sensitivity to small-scale keypoint regions.

#### 2.2.3. Multi-Head Self-Attention (MHSA) Module

In keypoint-based cattle body measurement tasks, the precise localization of specific anatomical landmarks is critical to ensuring measurement accuracy. For instance, the measurement of body length and cannon circumference relies on pairs of keypoints—either distant (as in body length) or relatively close (as in cannon circumference). In contrast, measurements such as withers height and hip height require only single-point localization. These varying spatial dependencies among keypoints impose greater demands on the network’s ability to model global contextual information effectively.

**Figure 3:**
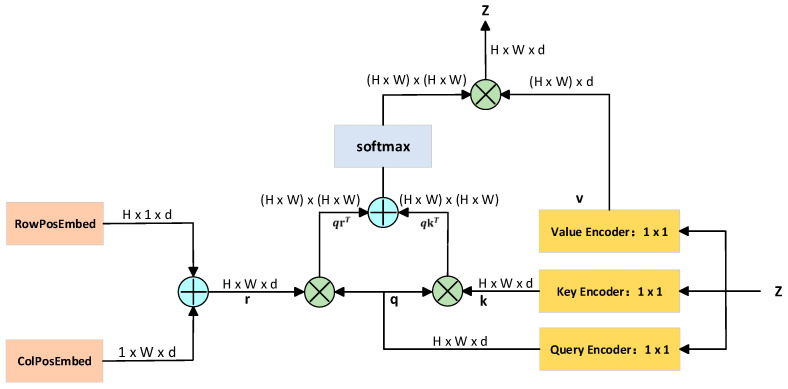
Architecture of the multi-head self-attention (MHSA) module.

The multi-head self-attention mechanism (MHSA) [27] first applies three independent linear projections to the input features to generate the query (Q), key (K), and value (V) vectors. Each attention head computes attention weights based on its own Q and K, which are then used to perform a weighted aggregation of the values across all positions, capturing contextual information of different semantic relationships. After parallel processing by multiple heads, their outputs are concatenated and fused through a linear transformation to produce new feature representations that encode global dependencies. This enhances the model’s ability to capture relationships between distant and local keypoints. The architecture of the MHSA module is illustrated in Figure 3.

In this study, the multi-head self-attention mechanism (MHSA) was combined with the lightweight C2f structure to construct a novel module, MHSA-C2f, which was integrated into the YOLOv8 network by inserting it before the detection head. This module enhances the model’s ability to capture spatial relationships of keypoints and global dependencies within the image, thereby effectively improving the accuracy and stability of keypoint detection. Such improvements meet the practical requirements of high-precision keypoint localization for fine-grained measurement of beef cattle body size.

#### 2.2.4. The Construction of the Fused Attention Module CASimAM

In the keypoint detection task addressed in this study, there are significant structural differences and spatial distribution characteristics among keypoints. On one hand, some keypoints are closely spaced with weak regional features; on the other hand, others are widely distributed, often located in the upper part of the target, making them more susceptible to occlusion and background interference—thus increasing localization difficulty. These challenges require the model not only to recognize locally salient regions but also to effectively model spatial structural relationships between keypoints. However, the original YOLOv8 backbone does not incorporate any explicit attention mechanisms, which limits the network’s ability to perceive discriminative keypoint regions. To address this, we introduced an attention module into the backbone stage to enhance the model’s responsiveness to keypoint-relevant regions, thereby improving overall detection accuracy and the precision of body size measurements.

To enhance the model’s ability to represent features in critical regions, this study integrates the SimAM (A Simple, Parameter-Free Attention Module) attention mechanism [28]. SimAM is a lightweight spatial attention module that introduces no additional parameters and incurs minimal computational cost, making it particularly suitable for deployment on resource-constrained edge devices, such as detection terminals in real livestock farms. This module is designed based on an energy function, performing point-wise saliency evaluation for all pixels within each channel to characterize their relative importance. The saliency of a given pixel is determined by its statistical relationship—specifically, the mean and variance—with other pixels in the same channel. Pixels with lower energy values are regarded as more discriminative and are assigned higher attention weights via a sigmoid activation function, thereby enhancing the corresponding feature responses.

**Figure 4:**
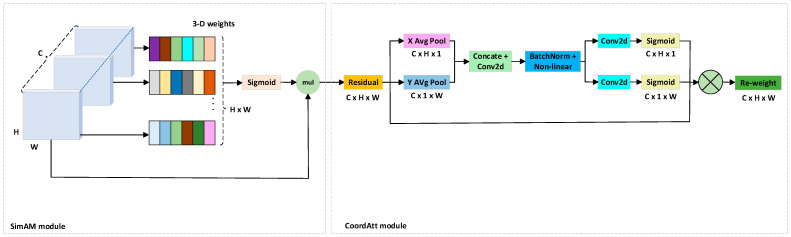
Structure of the CASimAM attention module.

After integrating the SimAM module into the backbone, the model’s perception of keypoint regions was notably enhanced, leading to improved localization accuracy. However, SimAM primarily focuses on pixel-level point-wise attention and lacks the ability to model spatial relationships and structural dependencies between keypoints. To further enhance the model’s awareness of spatial arrangements and fully exploit the complementary advantages of different attention mechanisms in keypoint modeling, we propose a novel attention module named CASimAM, which combines SimAM with Coordinate Attention. Specifically, the SimAM module first models pixel-level saliency within each channel, thereby enhancing the response to key regions in the feature map. Subsequently, the Coordinate Attention module [29,30] further explores spatial structural information through a decomposed coordinate attention mechanism. By encoding horizontal and vertical positional dependencies separately, Coordinate Attention enables the model to capture richer spatial structural relationships between keypoints. The two attention mechanisms complement each other: SimAM focuses on local saliency enhancement, while Coordinate Attention strengthens spatial orientation perception. Together, they significantly improve the model’s keypoint localization accuracy and structural understanding of cattle anatomy. The structure of CASimAM is illustrated in Figure 4.

To better align the proposed modules with the practical requirements of cattle body measurement, we emphasize their specific contributions to keypoint localization on the lateral view of cattle. The prediction of withers height, hip height, body length, and cannon circumference requires precise identification of anatomical landmarks such as the withers, hip, shoulder point, ischium, and forelimb cannon bone. In real farm environments, these landmarks are often subject to occlusion (e.g., limbs covering the trunk), illumination variation, or background clutter. The integration of DRAMiTransformer enhances long-range dependency learning, which improves robustness when landmarks such as the shoulder point or ischium are partially occluded. MHSA-C2f enhances feature discrimination in complex backgrounds and variable lighting, thereby stabilizing the localization of boundary points such as the withers and hip. CASimAM effectively emphasizes anatomically relevant areas—including the withers, hip, and cannon bone—while suppressing irrelevant background features. By addressing these challenges, the combined attention mechanisms significantly improve the accuracy and reliability of lateral-view cattle body measurement.

**Figure 5:**
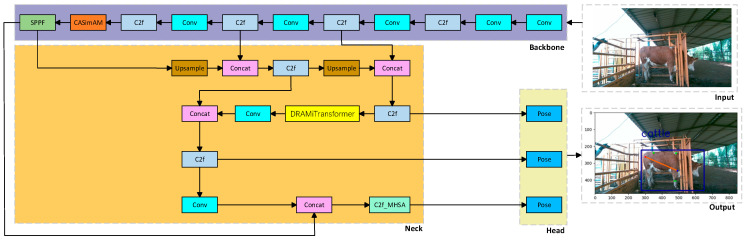
Architecture of the improved YOLOv8n model.

The enhanced YOLOv8-pose network, optimized in three key modules, not only improves the model’s capability to recognize cattle features in challenging environments and increases keypoint detection accuracy but also streamlines computational efficiency, making it highly suitable for edge computing devices. Utilizing content-aware upsampling and an efficiency-focused backbone, the model operates effectively even on resource-limited hardware, supporting real-time cattle monitoring and precise body dimension keypoint measurement. The architecture of the improved YOLOv8-pose network is presented in Figure 5.

### 2.3. Cattle Body Measurement Module

Although the collected RGB and depth images share the same resolution and are pixel-wise aligned, the depth map is often affected by lighting changes and environmental noise during acquisition. This may result in missing values at certain pixel locations in the depth image. When projecting pixels from the RGB image onto the depth map to obtain their corresponding 3D coordinates, the absence of valid depth values at those pixels prevents the calculation of accurate 3D information.

To address the issue of missing depth values caused by environmental interference or sensor errors, this study proposes a depth completion method based on the mean of the peripheral 16-neighborhood. When the depth value at a given pixel(i,j), denoted as $D\left(i,j\right)\;=\;0$, a local 5×5 window centered at this pixel is constructed. The window is defined as shown in Equation (1).

(1) $$\Omega_{ij}=\{(m,n)\;|\;i-2\leq m\leq i+2,\;j-2\leq n\leq j+2\}$$

From this window, the 16 pixels located on the outer boundary are extracted to form the neighborhood. The mean of the valid depth values in is then computed and used to fill the missing value at (i,j). This method effectively completes sparse depth regions, such as around the cattle’s edges or between the front and hind limbs, thereby improving the continuity of the depth map and enhancing the completeness and accuracy of the resulting 3D point cloud.

To eliminate background noise and emphasize the target region, a pass-through filtering process was applied to the optimized point cloud data [31,32]. Considering that the cattle body is primarily distributed along the direction of the optical center of the depth camera, the *Z*-axis is the spatial distribution that is most concentrated in this direction. Therefore, a threshold range is defined along the *Z*-axis based on the spatial distance between the camera and the cattle, in order to remove invalid points with depth values exceeding 5 m. The computation of the pass-through filter is defined as Equation (2).

(2) $$f(x,y,z)=\{(x,y,z)|z\in (z_{1},z_{2})\}$$

In this study, the improved YOLOv8-DMC model was employed to accurately detect key anatomical landmarks on the lateral images of cattle, including major body dimension parameters such as withers height, hip height, cannon girth, and body length. For each predicted keypoint, its 2D pixel coordinates (u,v) were first extracted from the RGB image, and the corresponding depth value Z was obtained from the aligned depth map.

**Figure 6:**
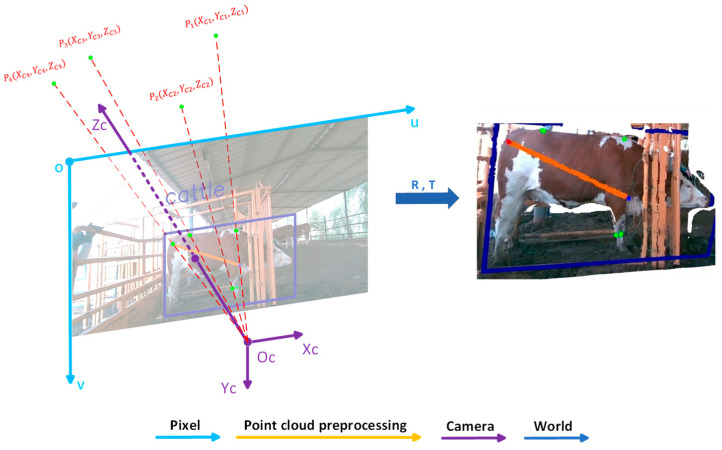
Conversion from 2D image coordinates to 3D world coordinates.

Based on the pinhole camera model and the intrinsic parameters of the camera $\left(f_{x},\;f_{y},\;c_{x},\;c_{y}\right)\;=\;\left(525,\;525,\;319.5,\;239.5\right)$, the image coordinates can be reprojected into the 3D camera coordinate system. The corresponding computation is shown in Equation (3).

(3) $$X=\frac{(u-c_{x})\cdot Z}{f_{x}},Y=\frac{(v-c_{y})\cdot Z}{f_{y}},Z=Z$$

where (u,v) denotes the pixel coordinates of the keypoint in the RGB image, and Z represents the depth value at the corresponding pixel in the depth map. Finally, by applying a spatial transformation matrix composed of a rotation (R) and translation (T), the coordinates in the camera coordinate system are transformed into world coordinates. A visual illustration of the transformation from 2D image coordinates to world coordinates is provided in Figure 6.

Based on the 3D coordinates of key points, precise spatial calculations were performed for various body measurements according to the established body dimension definitions and measurement benchmarks specified in the data annotation. Based on the 3D coordinates of the highest point at the bovine withers, the withers height was estimated by calculating the vertical distance to the ground. This was achieved using point clouds from hoof-adjacent regions in the depth map as a reference ground plane. The withers height was defined as the *Z*-axis coordinate difference between this key point and the ground plane. Based on the 3D coordinates of the highest point on the dorsal ridge at the anterior edge of the hip bone, the hip height was calculated as the vertical distance to the ground reference plane. This point—typically located slightly posterior to the sacral ridge—was precisely spatially localized through a detection model integrated with depth back-projection, ensuring the metric’s spatial accuracy. Body length was defined as the linear distance between the point of shoulder and the posterior edge of the pin bone. The spatial distance between these two key points was computed using the 3D Euclidean distance formula as shown in Equation (4).

(4) $$L=\sqrt{{\left(X_{1}-X_{2}\right)}^{2}+{\left(Y_{1}-Y_{2}\right)}^{2}+{\left(Z_{1}-Z_{2}\right)}^{2}}$$

The canonical circumference was estimated by detecting bilateral symmetric points at the narrowest tibial region of the bovine forelimb. Assuming an approximately circular cross-section in practical measurement, the perimeter was calculated using the three-dimensional Euclidean distance between these two points as the diameter.

### 2.4. Evaluation Metrics

In keypoint detection, the fundamental concept behind evaluation metrics draws inspiration from those used in object detection, where intersection over union (IoU) serves as a standard metric to quantify the overlap between predicted and actual targets. Similarly, keypoint detection employs object keypoint similarity (OKS) as the primary evaluation metric. The OKS is computed using the formula presented in Equation (5).

(5) $$OKS=\frac{\sum_{i}\left[exp\left(-\frac{d_{i}^{2}}{2s_{p}^{2}\sigma_{i}^{2}}\right)\cdot \delta (v_{i}>0)\right]}{\sum_{i}\left[\delta (v_{i}>0)\right]}$$

where $d_{i}$ represents the Euclidean distance between the predicted and ground-truth positions of the *i*th keypoint. $s_{p}$ denotes the area of the object’s bounding box, while $\sigma_{i}$ stands for the standard deviation reflecting the labeling accuracy for the *i*th keypoint. The term $v_{i}$ indicates the visibility status of that keypoint.

To evaluate model performance, the average precision (AP) is computed based on the object keypoint similarity (OKS). A threshold T is defined such that a keypoint is considered correctly predicted if its OKS exceeds T. For example, $AP_{@0.5}$ denotes the AP when the threshold is 0.5, while AP@[0.50:0.95] represents the mean AP over OKS thresholds ranging from 0.50 to 0.95 in steps of 0.05. The AP values are derived using Equation (6). Subsequently, the mean average precision (mAP) is obtained by averaging the AP values across all categories, serving as the final evaluation metric for keypoint detection. The calculation of mAP is shown in Equation (7).

(6) $$AP=\frac{\sum_{m}\sum_{p}OKS}{\sum_{m}\sum_{p}1}\left(OKS=\left\{\begin{array}{ll} OKS & \left(OKS>T\right)\\ 0 & \left(OKS\leq T\right) \end{array}\right.\right)$$

(7) $$mAP=\frac{\sum_{i=1}^{N}AP_{i}}{N}$$

In addition, precision (*P*) denotes the proportion of correctly predicted keypoints among all the keypoints predicted by the model, while recall (*R*) denotes the proportion of successfully detected keypoints among all the actual keypoints. Specifically, True Positive (*TP*) refers to keypoints correctly predicted by the model; False Positive (*FP*) refers to keypoints mistakenly detected by the model; and False Negative (*FN*) refers to actual keypoints that are missed by the model. The calculation formulas for precision and recall are shown in Equation (8) and Equation (9), respectively.

(8) $$\mathrm{Precision}=\frac{TP}{TP+FP}$$

(9) $$\mathrm{Recall}=\frac{TP}{TP+FN}$$

## 3. Experimental Results and Analyses

### 3.1. Experimental Platform and Parameter Settings

The experiments in this study were conducted on a Windows 10 64-bit operating system with an NVIDIA GeForce RTX 3090 GPU (NVIDIA Corporation, Santa Clara, CA, USA), powered by dual Intel (R) Xeon (R) Gold 5218 CPUs @ 2.30 GHz (Intel Corporation, Santa Clara, CA, USA), and 192 GB of RAM (IEIT Systems Co., Ltd., Jinan, China). The network model was implemented in the Python 3.10.9 environment using the PyTorch 2.0.1 deep learning framework. For cattle object detection experiments, the model was trained on a custom cattle behavior dataset. The training parameters were configured as follows: batch size of 16, 200 training epochs, learning rate of 0.01, and SGD optimizer. GPU acceleration was automatically enabled by default.

### 3.2. Improved Results for YOLOv8-Pose

#### 3.2.1. Comparison Between YOLO-Pose Versions

To evaluate the practical efficacy of the proposed improved model for cattle keypoint detection, we conducted systematic comparisons against mainstream lightweight pose estimation models: YOLOv5-pose, YOLOv8s-pose, YOLOv11-pose, and the baseline YOLOv8n-pose. Comprehensive metrics including Precision, Recall, mean Average Precision (mAP), parameter count, and computational complexity (GFLOPs) were benchmarked, with experimental results detailed in Table 2.

As shown in the table, the proposed improved model, YOLOv8-DMC-pose, achieved the highest performance in keypoint detection metrics, with $AP_{@0.5}$ and AP@[0.50:0.95] reaching 0.931 and 0.868, respectively. Compared to the baseline model YOLOv8n-pose, this represents an improvement of 2.14% and 3.09%, respectively. Additionally, the proposed model outperformed both the larger YOLOv8s-pose and the lightweight YOLOv11-pose. Notably, although YOLOv11-pose has fewer parameters, its $AP_{@0.5:0.95}$ remains 1.8% lower than the proposed model, indicating superior performance of our method in structural modeling and fine-grained keypoint perception.

In terms of recall and precision, the improved model achieved 0.881 and 0.882, respectively, maintaining a high level of performance comparable to YOLOv8s-pose and YOLOv11-pose, and significantly outperforming YOLOv5-pose. This indicates that the proposed improvement modules effectively enhance detection accuracy without negatively impacting recall or confidence score outputs.

In terms of model complexity, the improved model has 3.44 M parameters and 9.4 GFLOPs of computation. Compared to the baseline YOLOv8n-pose, this represents an increase of only 0.35 M parameters and 0.9 GFLOPs. However, it remains significantly lighter than YOLOv8s-pose, which has 11.42 M parameters and 29.6 GFLOPs. Compared to the accuracy-suboptimal YOLOv11-pose, the improved model increases parameters by 0.82 M and computation by 2.8 GFLOPs, but achieves notable gains in $AP_{@0.5}$ and $AP_{@0.5:0.95}$. These results demonstrate that the proposed model achieves a better trade-off between detection accuracy and model complexity, maintaining lightweight characteristics suitable for deployment on edge computing devices.

**Figure 7:**
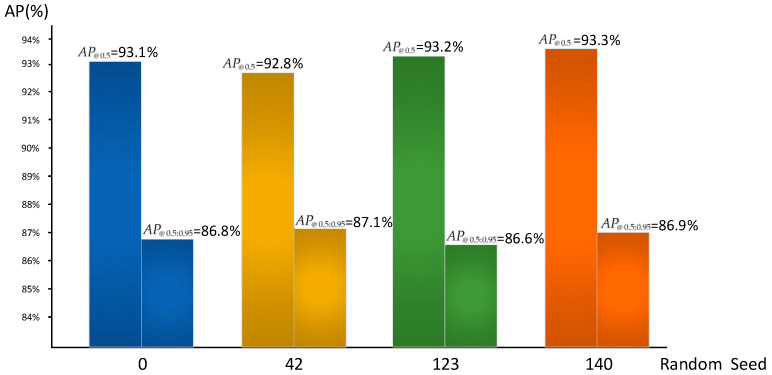
Performance of YOLOv8-DMC-pose under different random seeds.

To further evaluate the stability and robustness of the proposed model, we trained YOLOv8-DMC-pose under different random seeds (default, 42, 123, 140). As shown in Figure 7, the $AP_{@0.5}$ results (0.928–0.933) and $AP_{@0.5:0.95}$ results (0.866–0.871) remained highly consistent across runs, with variations within ±0.003, demonstrating reliable convergence and robustness of the model under different initialization settings. Instead of reporting per-keypoint AP, we follow standard pose estimation practices and adopt the overall mAP as a summary indicator, as it more directly reflects the end-to-end performance required in practical deployment scenarios. In addition, inference was benchmarked on an edge device (NVIDIA Jetson Xavier NX, NVIDIA Corporation, Santa Clara, CA, USA), where YOLOv8-DMC-pose achieved 28.3 FPS (35.4 ms per frame), confirming its suitability for real-time applications in farm environments.

In summary, the proposed improved model significantly enhances keypoint detection accuracy while maintaining low computational cost. It is particularly well suited for real-world scenarios such as ranches and livestock farms, where edge computing capability is limited. This provides a more reliable technical foundation for downstream applications such as cattle body size measurement.

#### 3.2.2. Ablation Experiment

To evaluate the impact of the proposed DRAMiTransformer, MHSA-C2f, and CASimAM modules on the YOLOv8n-pose network for keypoint detection in dairy cattle body measurement tasks, a series of ablation experiments were conducted. The results are presented in Table 3.

First, after integrating the DRAMiTransformer module into the backbone of YOLOv8n, the model’s $AP_{@0.5}$ increased to 0.9131, and $AP_{@0.5:0.95}$ reached 0.8440, representing improvements of 0.35% and 0.69%, respectively, compared to the baseline model (YOLOv8n). This demonstrates that the DRAMiTransformer effectively enhances the model’s ability for contextual modeling and structural awareness of keypoints.

Subsequently, with the addition of the MHSA-C2f module, which expands the receptive field and fuses multi-scale spatial attention, the $AP_{@0.5}$ improved to 0.9101 and $AP_{@0.5:0.95}$ to 0.8362, showing an increase of 0.05% and a slight decrease of 0.11%, respectively, indicating that this module contributes positively to spatial feature aggregation.

When using the CASimAM module alone, the model achieved an $AP_{@0.5}$ of 0.8973 and $AP_{@0.5:0.95}$ of 0.8342, slightly lower than the baseline (decreasing by 1.35% and 0.29%, respectively). However, when combined with the DRAMiTransformer, the $AP_{@0.5}$ improved to 0.9083 and AP@[0.50:0.95] to 0.8401, demonstrating that CASimAM serves as a beneficial complement in fine-grained spatial attention guidance. Furthermore, combining DRAMiTransformer with MHSA-C2f resulted in an $AP_{@0.5}$ of 0.9223 and AP@[0.50:0.95] of 0.8470, showing an overall improvement of 1.27% and 0.99% over the baseline, and outperforming other two-module combinations.

When all three modules were simultaneously embedded into the network structure (YOLOv8-DMC-pose), the performance reached its best: $AP_{@0.5}$ increased to 0.9310 and AP@[0.50:0.95] to 0.8680, improving by 2.14% and 3.09%, respectively, compared to the original YOLOv8n. Despite the significant accuracy gains, the parameter count only increased by approximately 0.35 M (from 3.09 M to 3.44 M), and GFLOPs grew by less than 0.9 (from 8.5 to 9.4). The model thus maintains a lightweight structure, making it well-suited for practical cattle body measurement scenarios requiring high precision and compatibility with edge deployment in pasture environments.

In summary, the proposed modules exhibit strong complementarity in spatial modeling, attention guidance, and feature representation. They effectively enhance the robustness and accuracy of dairy cattle keypoint detection in complex farm environments, providing a reliable foundation for subsequent body size estimation.

#### 3.2.3. Keypoint Detection Results

**Figure 8:**
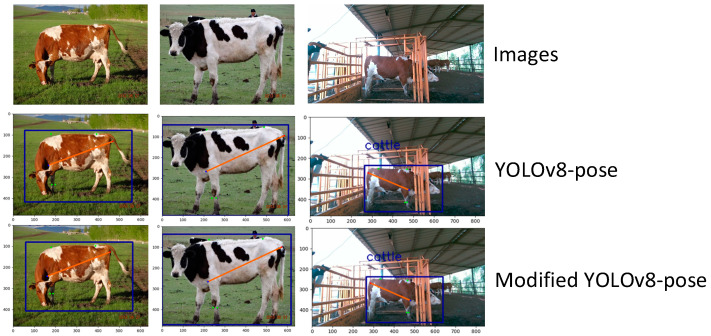
Keypoint prediction results.

To further evaluate the keypoint detection capability of the model in real-world farm environments, we selected several side-view images of cattle under varying background and lighting conditions. A comparative analysis was conducted between the original YOLOv8n-pose and the proposed improved model. As shown in Figure 8, both models were able to predict reasonable keypoints across most body regions. However, the improved YOLOv8n-pose demonstrated superior fitting accuracy, with predicted keypoints more closely aligned with manually annotated positions—particularly in areas such as leg joints and the dorsal contour—indicating stronger structural awareness and prediction stability. In contrast, the original model showed notable inaccuracies in specific keypoints, such as large deviations in the prediction of the cannon girth, and visible errors in key body size landmarks like height at withers and body length in certain samples.

### 3.3. Body Measurement Results

**Figure 9:**
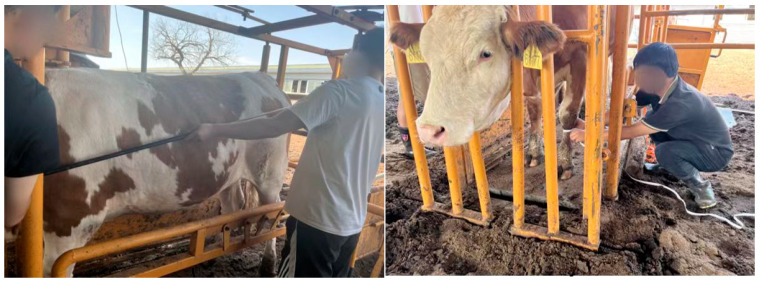
Manual body size measurement.

To obtain accurate and reliable body measurement data of cattle, individuals in a standard standing posture with an unobstructed side view were selected for manual measurements. All measurements were performed by experienced personnel under consistent environmental conditions. To minimize human error, each cow was measured 10 times, and the average value was taken as the final reference. Body height, withers height, and body length were measured using a livestock-specific measuring stick, while cannon circumference was measured with a soft tape measure. To ensure consistency in measurement standards, all operations were conducted by the same person. The manual measurement values served as the ground truth for evaluating the accuracy of the prediction algorithms. The actual measurement setup is shown in Figure 9.

Table 4 compares the manual body measurements of cattle with the algorithm’s predicted values. In the table, GT represents the ground truth (manual measurement), Pred denotes the model’s prediction, and RE is the relative error between the two. The average relative error for body height is 2.43%, mainly due to variations in cattle posture causing some deviation in the positioning of the withers keypoint. The average relative error for hip height is 2.26%, which is attributed to frequent bending of the hind limbs during behaviors such as defecation, affecting keypoint extraction accuracy. The average relative error for body length is 3.65%; larger errors in some measurement points are caused by posture changes during defecation, interfering with the extraction of the ischial tuberosity keypoint. The relative error for cannon circumference reaches up to 4.48%, primarily due to overlapping front limbs in certain postures, making it difficult for the keypoint detection network to precisely locate the narrowest part of the front limb’s tibia.

## 4. Discussion

In the task of keypoint prediction for lateral-view images of yellow cattle, the proposed improved YOLOv8n-pose algorithm achieved significant gains in both $AP_{@0.5}$ and AP@[0.50:0.95] indicating superior performance in localizing key body measurement points. This improvement is crucial for achieving accurate and reliable estimation of cattle body dimensions. Higher keypoint detection accuracy contributes to more robust and consistent predictions of key body parameters such as height, body length, and cannon girth. Notably, despite structural enhancements, the parameter size of the proposed model increased by only about 0.4 M compared to the original YOLOv8n, maintaining its lightweight nature. This balance between accuracy and computational efficiency makes the model well-suited for deployment on resource-constrained edge devices or in real-time on-site applications in farm environments. To address the issue of sparse or missing point clouds caused by lighting changes and background noise in complex farm settings—especially depth voids frequently observed on the cattle’s back—the study employed a depth completion strategy based on the mean of a 16-neighbor peripheral region. This approach effectively restores missing regions in the point cloud, ensuring that the keypoints predicted by the pose model can be matched with valid depth values. Without this completion step, 2D keypoints cannot be accurately projected into 3D world coordinates, which would compromise the precision of body measurement. On top of the completed point cloud, a pass-through filtering method was further applied to suppress background interference and enhance the usability of the point cloud data. By retaining only the structural information of the cattle body, this preprocessing step significantly improved the structural integrity and surface continuity of the 3D reconstruction results. Consequently, the accuracy and reliability of the final body dimension calculations were effectively enhanced.

Compared with typical error levels reported for single-view cattle body measurement, the results of this study are competitive. Previous approaches often showed relative errors above 5% for traits such as body length and height, particularly under complex farm conditions. In contrast, our method achieved 2.43% for body height, 2.26% for hip height, 3.65% for body length, and 4.48% for cannon circumference. These results suggest that the proposed YOLOv8-DMC framework provides higher accuracy and greater robustness while maintaining computational efficiency.

Despite these promising results, several limitations should be acknowledged. First, the validation was based on a relatively small dataset of 120 cattle, which may not fully capture breed variability and diverse farm conditions. Second, the system relies on strictly lateral views; deviations from this viewpoint could affect the accuracy of keypoint localization and subsequent measurements. Finally, the cannon circumference is partially occluded in many side-view images, which may limit precision compared with other traits. Addressing these limitations through larger, more diverse datasets and multi-view integration will be an important direction for future work.

## 5. Conclusions

To address the challenge of insufficient keypoint prediction accuracy in cattle body measurement, this study proposed a structural optimization of the YOLOv8n-pose model. Experimental results demonstrate that the proposed method significantly improves keypoint detection accuracy in side-view images of yellow cattle, with $AP_{@0.5}$ reaching 0.9310 and AP@[0.50:0.95] reaching 0.8680—representing improvements of 2.14% and 3.09%, respectively, over the original model. While maintaining a lightweight design, the model’s parameters increased by only 0.4 M, and the growth in computational complexity was limited to less than 0.9 GFLOPs, indicating strong deployment flexibility on resource-constrained edge computing devices.

The proposed method enhances the accuracy of key measurement point localization while preserving efficiency and practicality, providing robust support for non-contact 3D cattle reconstruction and body dimension estimation. The findings offer valuable insights for precision management and health assessment of individual cattle in livestock farming, and contribute a novel approach to the field of automated animal body measurement.

Despite the promising results, limitations remain when handling complex postures—such as overlapping forelimbs—which may cause deviations in keypoint extraction and affect the accuracy of specific measurements. Future research will focus on enhancing the model’s robustness and adaptability in occluded or complex scenes, promoting its broader application in real-world farming environments. To address the keypoint extraction deviation caused by overlapping forelimbs, future research will introduce a cattle behavior detection algorithm: by leveraging the RGB/depth data from the depth camera used in this study, images containing overlapping forelimbs will be filtered out, and only images with standard postures will be retained for key point detection in cattle body measurement—thus reducing keypoint errors at the source. In comparison with existing pose estimation approaches for livestock, our method achieves higher accuracy while maintaining computational efficiency, highlighting its novelty and practical advantages despite certain limitations. Moreover, the proposed non-contact measurement method holds promise for practical applications in livestock management, including breeding evaluation, weight prediction, and health monitoring. To further support reproducibility, future work will also consider open-sourcing the trained model and annotation protocol, enabling other researchers to validate and extend our findings under diverse farming conditions.

## Figures and Tables

**Table 1:** Dataset composition and split ratio.

Subset	Number of Images	Ratio (%)
Training	4954	70
Validation	1415	20
Test	708	10

**Table 2:** Model evaluation results for different versions.

Model	Precision	Recall	$AP_{@0.5}$	AP@[0.50:0.95]	Parameters (M)	GFLOPs
YOLOv5-pose	0.8756	0.8850	0.8877	0.8298	3.09	8.5
YOLOv8n-pose	0.8891	0.8753	0.9096	0.8371	3.09	8.5
YOLOv8s-pose	0.8970	0.8750	0.9100	0.8420	11.42	29.6
YOLOv11-pose	0.8986	0.8868	0.9117	0.8500	2.62	6.6
YOLOv8-DMC-pose	0.8820	0.8810	**0.9310**	**0.8680**	3.44	9.4

**Table 3:** Results of ablation experiments.

Model	Precision	Recall	$AP_{@0.5}$	AP@[0.50:0.95]	Parameters (M)	GFLOPs
YOLOv8n	0.8891	0.8753	0.9096	0.8371	3.09	8.5
YOLOv8n + DRAMiTransformer	0.8990	0.8731	0.9131	0.8440	3.13	8.9
YOLOv8n + MHSA-C2f	0.8761	0.8773	0.9101	0.8362	3.14	8.5
YOLOv8n + CASimAM	0.8902	0.8760	0.8973	0.8342	3.10	8.5
YOLOv8n + DRAMiTransformer + MHSA-C2f	0.8895	0.8856	0.9223	0.8470	3.18	9.0
YOLOv8n + DRAMiTransformer + CASimAM	0.9021	0.8712	0.9083	0.8401	3.14	9.0
YOLOv8-DMC-pose	0.8820	0.8810	**0.9310**	**0.8680**	3.44	9.4

**Table 4:** Comparison with traditional measurement methods (N = 10).

Cattle ID	Body Height (cm)	Hip Height (cm)	Body Length (cm)	Cannon Circumference (cm)
	GT/Pred/RE (%)	GT/Pred/RE (%)	GT/Pred/RE (%)	GT/Pred/RE (%)
1	125/126.5/1.20%	132.5/134.2/1.31%	139.5/137.1/1.78%	19.5/19.9/2.36%
2	143/141.8/0.86%	145.5/147.2/1.22%	165/168.5/2.11%	21/21.6/2.94%
3	138.5/141.4/2.10%	146.5/145.1/0.98%	173.5/171.2/1.30%	22/22.7/3.45%
4	134/131.3/1.98%	136/137.2/0.86%	173.5/177.9/2.56%	18/17.5/2.77%
5	131.5/134.5/2.34%	134.2/136.2/1.49%	150/152.8/1.92%	20/19.2/3.90%
6	146/147.4/0.97%	150/151.7/1.16%	170/172.4/1.45%	23/23.7/3.12%
7	131.5/132.7/0.90%	134.2/137.6/2.56%	149.5/151.9/1.63%	20/20.8/4.10%
8	125.5/124.2/1.10%	132/135.1/2.39%	146/143.5/1.72%	20/19.3/3.56%
9	138/141.4/2.50%	142/143.6/1.10%	173/169.8/1.86%	24/25/4.54%
10	140/142/1.45%	146/144.2/1.25%	151/153.7/1.77%	20.5/21.1/2.97%

## Data Availability

Informed consent was obtained from the farm owners/managers for the use of cattle images collected during routine herd management.
